# A cross-sectional analysis of association between visceral adiposity index and serum anti-aging protein Klotho in adults

**DOI:** 10.3389/fendo.2023.1082504

**Published:** 2023-02-06

**Authors:** Jianwei Cui, Zhenzhen Yang, Jiahao Wang, Shan Yin, Yunfei Xiao, Yunjin Bai, Jia Wang

**Affiliations:** ^1^ Department of Urology, Institute of Urology, West China Hospital, Sichuan University, Chengdu, China; ^2^ Department of Clinical Laboratory, Nanchong Central Hospital, Nanchong, China; ^3^ Department of Urology, Affiliated Hospital of North Sichuan Medical College, Nanchong, China

**Keywords:** visceral adiposity index, Klotho, obesity, aging, NHANES

## Abstract

**Background:**

The visceral adiposity index (VAI) is regarded as a reliable indicator to assess body fat distribution and dysfunction. Klotho protein is a hormone with anti-aging biological functions. However, the relationship between them has not been researched.

**Objects:**

This study aimed to evaluate the association between VAI and serum anti-aging protein klotho in American adults.

**Methods:**

A cross-sectional study of participants was conducted based on the National Health and Nutrition Examination Surveys (NHANES) 2007–2016. Visceral adiposity was determined using the VAI score, while the klotho protein concentration was measured by ELISA kit. After adjusting some possible confounding variables, multivariate regression model was conducted to estimate the relationship between VAI and klotho protein. Furthermore, the smooth curve fitting and the segmented regression model were applied to examine the threshold effect and to calculate the inflection point.

**Result:**

In total, 6 252 adults were eligible, with a mean VAI of 2.04 ± 0.03 and a mean klotho protein concentration of 848.79 ± 6.98 pg/ml. Multivariate regression analysis indicated that serum klotho protein concentration was lower in participants with high VAI score. When VAI was divided into quartiles, participants in the fourth quartiles of higher VAI had lower klotho protein levels (Q4: -32.25 pg/ml) than participants in the lowest quartile (Q1) after full adjustment (P < 0.05). Segmented regression suggested that the turning point value of VAI was 3.21. A 1-unit increase in VAI was significantly associated with lower klotho protein levels by -18.61 pg/ml (95% CI: -28.87, -8.35; P < 0.05) when VAI ranged from 0.29 to 3.21(accounting for 83.7% of the participants), however, the association was not significant when VAI ranged from 3.21 to 11.81 (P = 0.77).

**Conclusion:**

There was a nonlinear correlation between VAI score and the serum anti-aging protein klotho concentrations, showing a saturation effect. When VAI was less than 3.21, they were negatively correlated, and when VAI was greater than 3.21, they had no obvious correlation.

## Introduction

With the changes in dietary nutrition structure and daily lifestyle, the prevalence of being overweight or obese has been increasing rapidly around the world (1). Obese individuals, especially those with excessive abnormal fat accumulation, are more susceptible to type 2 diabetes, hypertension, and atherosclerotic events, which affect human life span (2, 3). The visceral adiposity index (VAI) is an indicator that can reliably evaluate visceral fat distribution and function in adults (4). Compared with traditional adiposity indexes, such as body mass index (BMI) and waist circumference (WC), VAI has a better predictive capacity and stronger correlation with unhealthy metabolic phenotypes (5). Sensitive detection methods for visceral obesity, including computed tomography and magnetic resonance imaging, are characterized by high costs, time consumption and radiation hazards, making these techniques unsuitable for large-scale populations (6). VAI, which includes not only anthropometric (BMI and WC), but also metabolic parameters [triglyceride (TG) and high-density lipoprotein (HDL)], is regarded as a more easily applicable and reliable indicator to assess body fat distribution and dysfunction. Studies have shown that VAI, associated significantly with cardiovascular events and atherosclerosis, is also an independent risk factor for coronary artery disease, hypertension and diabetes (7).Increased insulin resistance and low-grade chronic inflammation, caused by an increase in visceral adipose tissue, are thought to be a possible cause of metabolic diseases (8).

Klotho protein, encoded by the klotho gene, possesses remarkable anti-aging abilities. Three members of the klotho family have been identified: α-, β-, and γ-isoforms (9). α-klotho acts as an obligate coreceptor for fibroblast growth factor 23, and can be released from cells into the blood after cleaved by secretases (10). Its transgenic overexpression has been revealed to extend lifespan up to 30% in transgenic mice (11). On the contrary, mice homozygous for mutations in the klotho gene exhibit many pathways of the aging process including skin atrophy, shortened lifespan, growth retardation, arteriosclerosis, and osteoporosis (12, 13). The expression of klotho protein is beneficial to cardiovascular diseases by improving endothelial dysfunction and alleviating arteriosclerosis (14, 15). Meanwhile, downregulation of this protein was also reported to involve the common aging-related disorders, such as cancer, metabolic syndrome, and chronic kidney disease (13, 16, 17). Some of the above diseases are related to obesity, such as arteriosclerosis and osteoporosis (18, 19). In addition, reduced levels of klotho in white adipose tissue were found to be associated with high fat-induced obesity in non-human primates (20). And it has been consistently reported that serum klotho level was inversely correlated with age in humans (21). As one of the robust markers of biological aging, shorter leukocyte telomere length was reported to be related with higher VAI score (22). Nevertheless, little research has been reported on the direct relationship between VAI and serum klotho protein in humans.

Diets inducing weight loss through caloric restriction, could improve metabolism and increase lifespan (23). But the exact mechanism by which weight control affects longevity remains unclear. Klotho expression levels may potentially be involved in the relationship between adiposity obesity and aging. Therefore, we used large population data from the National Health and Nutrition Examination Survey (NHANES) to analyze the association between VAI and klotho protein, to provide new ideas for exploring the mechanism. We hypothesized that higher VAI was associated with lower serum klotho protein concentrations.

## Participants and methods

### Study design and population

This study information is based on NHANES 2007–2016. NHANES is a program of studies aiming to investigate the health and nutritional status of American participants, and involves interviews, examinations, and laboratory components. The data consisted of 5 continuous cycles from 2007 to 2016 (other cycles did not include VAI or klotho protein). Participants who were pregnant (n=317) were excluded from the total included population (n=50588). Next, 43891 participants without data information of VAI or klotho protein were also excluded from the study. After the sensitivity analysis performed by removing extreme values(VAI>99% percentage or <1% percentage, n=128), 6252 eligible participants were included for further analyses. All the study protocols included here obtained written informed consent and were approved by the Research Ethics Review Board at the National Center for Health Statistics.

### Outcome and exposure factors

The major exposure factor was VAI, calculated using the following sex-specific formula (24): Males: VAI = {WC/[39.68+(1.88*BMI)]} * (TG/1.03) * (1.31/HDL);

Females: VAI = {WC/[36.58+(1.89*BMI)]} * (TG/0.81) * (1.52/HDL).

WC is measured in cm, BMI in kg/m^2^, TG and HDL in mmol/L.

The main outcome was serum klotho concentration. Serum specimens from participants were collected, transferred and stored at -80°C. Serum klotho concentrations were measured by a commercially available ELISA kit produced by IBL International, Japan. The assay sensitivity was 6 pg/mL. All study samples were run in duplicate, bisected and measured separately, and the average of the two concentrations was calculated as the result.

### Covariates

Additional covariates were collected from each cycle of NHANES. The continuous variables included age, and poverty income ratio (PIR). Categorical variables included gender, age, race, education level, marital status, smoking and alcohol use. According to previous studies, several possible confounding variables were adjusted. Specifically, age was defined as 40–49, 50–59, and ≥60 years. BMI was divided into <25, 25–30, and ≥30 kg/m^2^. Participants were stratified by PIR: ≤1.3, >1.3 & ≤3.5, >3.5%, and missing. Race was classified as Mexican American, other Hispanic, non-Hispanic white, non-Hispanic black, and other races. Marital status was divided into married, widowed, divorced, separated, never married, and living with partner. Education level was divided into < 9th grade, 9–11th grade, high school graduate, some college, and college graduate or above. Physical activity was categorized as <500, ≥500 and missing. Smoking status included never (less than 100 cigarettes in lifetime), former (100 cigarettes or more but no currently smoking) and now (100 cigarettes or more and currently smoking), and alcohol consumption included <12 drinks/year, ≥12 drinks/year and missing. Participants with EGFR≤ 60 ml/min/1.73m^2^ or urinary albumin/creatinine ratio ≥ 30 mg/g were defined as having chronic kidney disease. Self-reported medical conditions included cancer, diabetes, hypertension, stroke, cardiovascular disease (coronary heart disease, angina, congestive heart failure), and chronic obstructive pulmonary disease (all classified as Yes/No). When the value of the missing covariable was more than 2% of the total population, dummy variables were used instead.

### Statistical analysis

The baseline characteristics of all participants are clearly described in **Table 1**, by the means of proportions or the mean ± standard error (SE). Particularly, categorical variables were presented by weighted chi-square analysis, and simultaneously, the continuous variables were evaluated by a weighted linear regression model. VAI was treated as not only a continuous independent variable, but also a categorical variable (divided into quartiles), with the lowest quartile used as the reference.

**Table 1:** Characteristics of participants by categories of visceral adiposity index (VAI) in NHANES 2007–2016^ab^.

Characteristic	All	VAI quartiles				P value
		Q1(0.29-0.95)	Q2(0.95-1.55)	Q3(1.55-2.56)	Q4(2.56-11.81)	
N. of participants	6252	1563	1563	1563	1563	
Visceral adiposity index	2.04 ± 0.03	0.67 ± 0.01	1.23 ± 0.01	2.02 ± 0.01	4.24 ± 0.07	<0.0001
Klotho (pg/ml)	848.79 ± 6.98	867.62 ± 10.82	867.55 ± 11.14	835.83 ± 9.42	820.42 ± 11.80	<0.0001
Age (years)	56.30 ± 0.20	56.03 ± 0.47	55.84 ± 0.43	56.91 ± 0.27	56.47 ± 0.30	0.0103
40-49 (%)	31.22	33.50	33.07	27.72	30.54	
50-59(%)	30.87	26.30	31.38	34.56	30.87	
≥60 (%)	37.92	40.20	35.55	37.72	38.58	
Gender (%)						0.0145
Female	52.45	52.76	49.34	56.91	52.30	
Male	47.55	47.24	50.66	43.09	47.70	
Race (%)						<0.0001
Mexican American	6.44	4.34	5.36	8.86	7.31	
Other Hispanic	4.94	3.81	5.10	5.41	5.38	
Non-Hispanic white	73.78	71.69	74.02	72.40	77.34	
Non-Hispanic black	8.55	13.51	9.40	6.99	4.01	
Other races	6.29	6.65	6.13	6.34	5.97	
Poverty income ratio	3.25 ± 0.06	3.50 ± 0.08	3.35 ± 0.07	3.10 ± 0.07	3.05 ± 0.08	<0.0001
≤1.3 (%)	16.40	13.32	15.68	17.31	19.17	
>1.3 and ≤3.5 (%)	31.07	27.55	28.52	34.87	33.61	
>3.5 (%)	46.33	53.03	49.49	41.13	41.73	
Missing (%)	6.20	6.10	6.32	6.70	5.49	
BMI (kg/m^2^)	29.51 ± 0.15	26.74 ± 0.20	28.68 ± 0.24	31.01 ± 0.26	31.83 ± 0.18	<0.0001
<25 (%)	24.43	43.84	26.96	15.96	9.73	
25-30 (%)	35.73	34.18	41.11	33.67	33.93	
≥30 (%)	39.85	21.98	31.93	50.37	56.34	
Education (%)						<0.0001
Less than 9th grade	6.22	4.94	5.61	7.21	7.02	
9-11th grade	10.48	7.71	9.88	11.45	12.71	
High school graduate	21.31	17.58	21.19	22.38	24.66	
Some college	30.23	27.13	30.66	31.58	32.24	
College graduate or above	31.77	42.64	32.67	27.38	23.37	
Marital Status (%)						0.3608
Married	66.21	69.72	65.51	65.02	64.91	
Widowed	5.79	4.79	5.71	5.78	7.17	
Divorced	13.51	11.11	15.18	13.18	13.79	
Separated	2.23	2.22	2.01	2.41	2.30	
Never married	7.46	7.60	6.77	8.52	6.97	
Living with partner	4.80	4.56	4.82	5.09	4.86	
Smoking (%)						<0.0001
<100 cigarettes in life	51.23	58.25	51.23	51.50	44.27	
≥100 cigarettes in life but no smoking now	30.83	28.54	29.80	32.23	32.89	
≥100 cigarettes while smoking now	17.94	13.22	18.97	16.27	22.84	
Alcohol (%)						0.0048
<12 drinks/year	22.03	19.30	21.62	22.68	25.53	
≥12 drinks/year	72.52	73.72	72.79	71.69	70.87	
Missing	5.45	6.98	5.59	5.63	3.59	
Physical activity(%)						<0.0001
<500	14.07	13.77	12.86	14.87	15.37	
≥500	61.77	69.61	66.02	55.72	54.78	
Missing	24.17	16.62	21.12	29.42	29.86	
Chronic kidney disease (%)						<0.0001
No	84.72	88.62	88.54	83.67	78.82	
Yes	15.28	11.38	11.46	16.33	21.18	
Cancer (%)						0.1782
No	87.24	88.10	87.63	87.92	84.83	
Yes	12.76	11.90	12.37	12.08	15.17	
Diabetes (%)						
No	78.32	89.14	83.13	76.13	65.85	<0.0001
Yes	21.68	10.86	16.87	23.87	34.15	
Hypertension (%)						
No	51.40	63.67	55.44	48.25	38.81	<0.0001
Yes	48.60	36.33	44.56	51.75	61.19	
Stroke (%)						
No	96.35	96.90	96.41	96.75	95.22	0.1130
Yes	3.65	3.10	3.59	3.25	4.78	
Cardiovascular disease(%)						
No	88.57	90.77	90.77	87.49	85.63	0.0002
Yes	11.43	9.23	9.23	12.51	14.37	
Chronic obstructive pulmonary disease(%)						
No	92.55	93.98	92.06	92.00	92.00	0.3239
Yes	7.45	6.02	7.94	8.00	8.00	

a Mean ± SE for continuous variables, and P value calculated by weighted t test.

b % for categorical variables, and P value calculated by weighted Chi-square test.

To study the independent relationship between VAI and klotho protein, multivariate generalized linear regression analyses were conducted. Model 1 was adjusted for no covariate. Model 2 was adjusted for age, gender, and race. Model 3 was adjusted for gender, age, race, PIR, BMI, education, marital status, physical activity, smoking, alcohol use, chronic kidney disease, cancer, diabetes, hypertension, stroke, chronic obstructive pulmonary disease, and cardiovascular disease, for the purpose of further subgroup analysis.

The smooth curve fitting and a generalized additive model were set up in order to explore the potential non-linear correlation. We further applied the segmented linear regression model to examine the threshold effect and to calculate the inflection point.

We used sampling weights recommended by the CDC guidelines to account for the complex study design in all the analysis except for the curve fitting (25). All the above statistical analyses were completed by using R 3.6.3 and EmpowerStats. A P-value <0.05 was considered to be of statistical significance (two-tailed).

## Results

### Participant characteristics

The participants’ characteristics at the baseline according to the categories of VAI were presented in **Table 1**. A total of 6252 American adults were qualified to be enrolled in the study. Among all participants, 52.45% were females and 47.55% were males. The mean ± SE of VAI was 2.04 ± 0.03. The mean ± SE of klotho protein concentration was 848.79 ± 6.98 pg/ml. The serum klotho protein concentration in the last quartile was lowest (Q4: 820.42 ± 11.80 pg/ml), compared to the other three quartiles (Q1: 867.62 ± 10.82, Q2: 867.55 ± 11.14, Q3: 835.83 ± 9.42 pg/ml, p < 0.01).

### Multivariate regression analysis

**Table 2** demonstrated that VAI was negatively correlated with klotho protein concentration in the non-adjusted model [β(95%CI) = -12.95 (-18.95, -6.94)], minimally adjusted model [-12.22 (-18.45, -6.00)], and the fully adjusted model [-9.80 (-16.37, -3.22)]. Multivariate regression analysis indicated that serum klotho protein concentration was lower in participants with higher VAI score. When VAI was divided into quartiles, participants in fourth quartiles of VAI had lower klotho protein levels (Q4: -32.25 pg/ml), than participants in the lowest quartile (Q1) after full adjustment (P < 0.05). Although no negative significant difference was seen in the prevalence in the second quartile group, there was a significant relationship between VAI and klotho protein level in the fourth quartile group in all three models. Compared to participants with lower VAI (0.29–0.95) in the first quartile group, people with higher VAI (2.56–11.81) in the fourth quartile group, had a significantly lower level of klotho protein in model 1 [-47.20 (-75.94, -18.46)], model 2 [-42.20 (-71.62, -12.79)], and model 3 [-32.25 (-64.26, -0.25)]. P values for trend were less than 0.05 in all three models.

**Table 2:** Association between visceral adiposity index (VAI) and serum anti-aging protein klotho.

Exposure	Model 1[^a^]	Model 2[^b^]	Model 3[^c^]
	β(95% CI) P value	β (95% CI) P value	β (95% CI)P value
VAI	-12.95 (-18.95, -6.94)	-12.22 (-18.45, -6.00)	-9.80 (-16.37, -3.22)
	0.00006	0.00026	0.00436
VAI quartile			
Q1	Ref	Ref	Ref
Q2	-0.07 (-25.40, 25.27)	2.17 (-23.01, 27.35)	7.46 (-18.44, 33.36)
	0.99585	0.86626	0.56386
Q3	-31.79 (-58.20, -5.38)	-32.40(-59.22, -5.58)	-25.49 (-54.06, 3.08)
	0.02089	0.02070	0.07873
Q4	-47.20 (-75.94, -18.46)	-42.20 (-71.62, -12.79)	-32.25 (-64.26, -0.25)
	0.00189	0.00641	0.04818
VAI quartile continuous	-17.35(-26.74,-7.96)	-16.12 (-25.79, -6.46)	-13.05 (-23.57, -2.53)
	0.00052	0.00166	0.01611

a Model 1: adjusted for no covariates.

b Model 2: adjusted for age, gender, race.

c Model 3: adjusted for gender, age, race, poverty income ratio, body mass index, education, marital status, physical activity, smoking, alcohol use, chronic kidney disease, cancer, diabetes, hypertension, stroke, chronic obstructive pulmonary disease, and cardiovascular disease.

### Non-linear analysis

**Figure 1:**
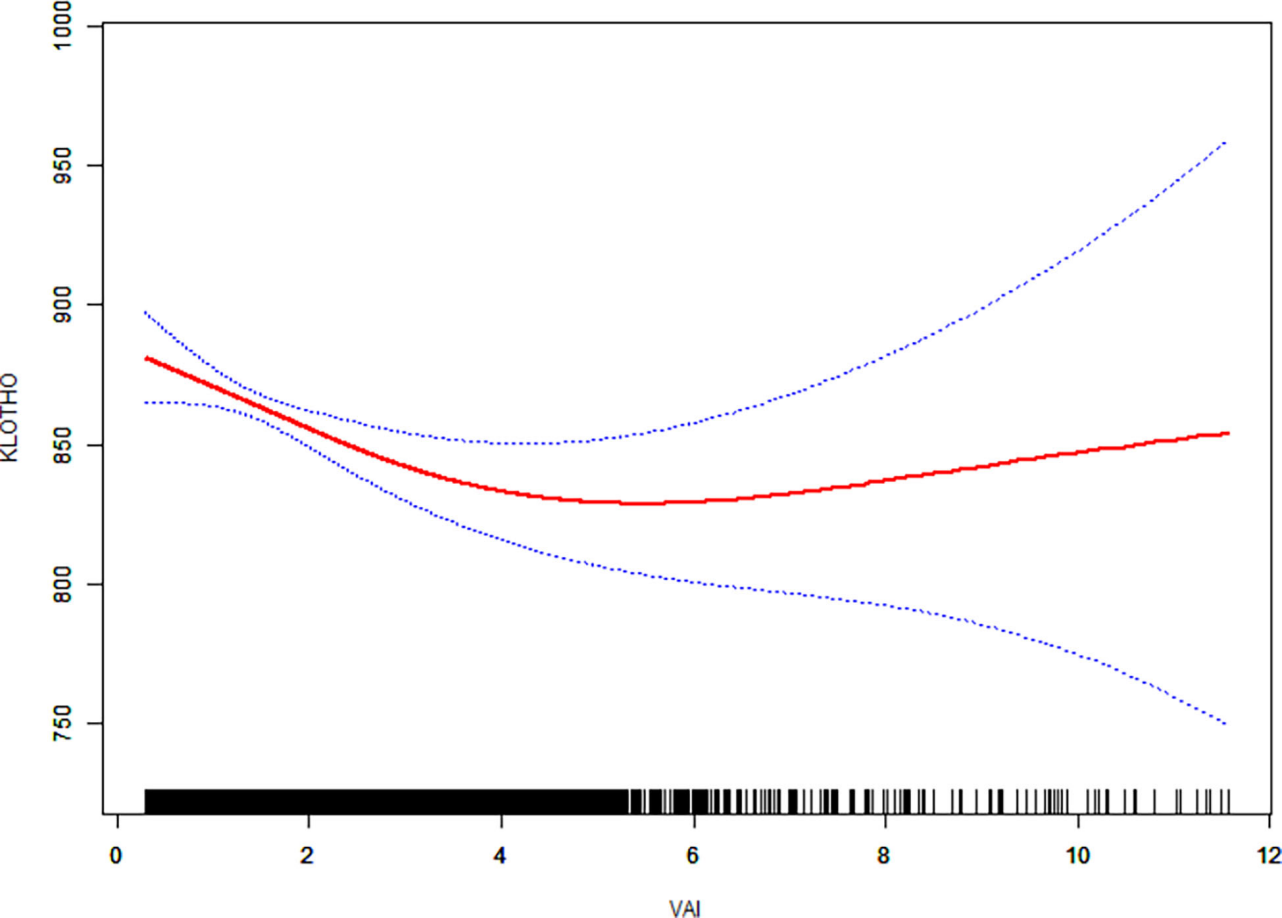
The relationship between visceral adiposity index (VAI) and serum anti-aging protein klotho. Solid line in the middle represents smooth curve fitting between variables. The dotted line on both sides represents a 95% confidence interval (CI) for the fit. The bottom black and white strip shows the population density according to VAI. All models were adjusted for gender, age, race, poverty income ratio, body mass index, education, marital status, physical activity, smoking, alcohol use, chronic kidney disease, cancer, diabetes, hypertension, stroke, chronic obstructive pulmonary disease, and cardiovascular disease.

We then performed a smooth curve fitting and a segmented regression to explore the non-linear association between VAI and serum klotho protein concentration (**Figure 1**; **Table 3**). The smooth curve after fully adjustment showed a non-linear relationship between VAI and klotho protein level (**Figure 1**). The segmented regression suggested that the turning point value of VAI was 3.21 (**Table 3**). A 1-unit increase in VAI was significantly associated with lower klotho protein levels by 18.61 pg/ml (95% CI: -28.87, -8.35; P < 0.01) when VAI ranged from 0.29 to 3.21 (5 233 individuals, accounting for 83.7% of the participants), however, the association was not significant when VAI ranged from 3.21 to 11.81 (P > 0.05, log likelihood ratio test = 0.017).

**Table 3:** Threshold effect analysis for the relationship between visceral adiposity index (VAI) and serum anti-aging protein klotho in NHANES 2007–2016^a^.

Model	serum klotho protein concentration	
	Adjusted β (95% CI)	P value
Model I		
the standard linear mode	-7.84 (-13.02, -2.65)	0.0030
Model II		
Turning point (K)	3.21	
VAI < 3.21 (accounting for 83.7% of the participants)	-18.61 (-28.87, -8.35)	0.0004
VAI > 3.21 (accounting for 16.3% of the participants)	1.40 (-7.79, 10.59)	0.7652
Log likelihood ratio test	0.017	

a All models were adjusted for gender, age, race, poverty income ratio, body mass index, education, marital status, physical activity, smoking, alcohol use, chronic kidney disease, cancer, diabetes, hypertension, stroke, chronic obstructive pulmonary disease, and cardiovascular disease.

## Discussion

To our knowledge, this is the first study to evaluate the relationship between VAI and serum anti-aging protein klotho concentration by analyzing a large population data in NHANES. There was a dose–response negative association: on the left side of the turning point, a higher VAI score was associated with a decreased level of serum klotho concentration. Further study demonstrated that klotho decreased 18.61 pg/ml for every 1 unit increase in VAI among Americans. However, on the right side of the turning point, the relationship was not significant.

Klotho is mainly expressed in kidney and brain tissues and has hormone-like functions. On the one hand, the anti-aging mechanism of klotho protein is related to the down-regulation of phosphate reabsorption and up-regulation of calcium reabsorption, thereby inhibiting the increase of vitamin D level (26). On the other hand, another mechanism is through inhibition of various Wnt ligands, of which overactivity is related to aging and tumorigenesis (27). Additionally, klotho can suppress inflammation and oxidative stress (28). In a recent study, negative associations were observed between α-klotho and some inflammatory markers (14). Therefore, this protein may play an important role in anti-aging through its anti-inflammatory effect.

According to 239 large, multinational, prospective studies performed in four continents, the relationship between overweight and obesity measured by BMI, and higher all-cause mortality was indisputable (29). Our study showed that visceral obesity measured by VAI was negatively correlated with anti-aging protein klotho. Obesity is characterized by changes in the distribution of adipose tissue and the mass expansion of the body. Obesity can be divided into visceral obesity, excess adipose tissue accumulated in the abdomen, and subcutaneous obesity, accumulation of adipose tissue under the skin (30). Furthermore, surgical removal of visceral fat in rats was proved to be beneficial for improving insulin sensitivity, reducing the incidence of liver and kidney disease, and extending lifespan (31, 32). These studies demonstrated that visceral fat accumulation made contributions to the reduction in life expectancy in aging.

Previous studies have demonstrated that visceral obesity is a risk factor for various metabolic diseases (33), while subcutaneous obesity does not appear to be related with those metabolic syndromes (34). A relationship has been suggested between the accumulation of abdominal fat and a low-grade elevation of inflammatory mediators, such as c-reactive protein, tumor necrosis factor, in the circulating concentrations in the body fluids (35). Normally, lymphatic tissue and the liver are the main producer of these inflammatory mediators, but in obesity, adipose tissue becomes the main site of production, leading to a long-term and sustained environment of local and systemic inflammation (30). Recent study has shown that not only local but also systemic inflammation could decrease klotho expression in the kidneys (36). Additionally, Ma et al. found that chronic inflammation, caused by a pro-inflammatory diet pattern, can reduce serum klotho levels (37). Thus, as a characteristic of the obese state, the excessive proinflammatory products, mainly from adipose tissue, may in turn deplete the klotho protein in the serum, reducing its concentration.

A compelling study suggested that diets inducing weight loss through caloric restriction, could improve metabolism and increase lifespan by positively affecting adipose tissue (23). In addition, white adipose tissue is the main component of visceral tissue and can secrete adipokines. The increase of visceral fat leads to the accumulation of triglycerides, resulting in the dysregulation of adipokine secretion (38). Adipokines affect immune cell chemotaxis and promote the release of inflammatory cytokines, which ultimately affect klotho protein synthesis and promote cellular aging (38). Besides, visceral adiposity has a positive correlation with oxidative stress and insulin sensitivity, while klotho expression has a negative relation with oxidative stress (6, 39). Thus, oxidative stress, caused by excess visceral fat, may deplete some of this protein. More studies are needed to discover the detailed mechanism.

Recently, it has been demonstrated for the first time that the relationship between cerebrospinal fluid α-klotho and BMI was inverse (40). Likewise, the same held true for the association between the serum α-klotho and VAI in our study. These two studies prove the correlation between obesity and klotho protein from different angles. This protein concentration is closely related to lipid levels: negatively related to TG and positively related to HDL (41, 42). In addition to these two parameters, the VAI score includes waist circumference and BMI, which quantifies the severity of visceral obesity. We also determined the optimal VAI cut-off value to clarify the relationship between VAI and klotho protein in different cases, which provides a new perspective for the study of visceral obesity and aging.

There are some strengths in our study. Firstly, as far as we were aware, this was the first report of an association between visceral adiposity and serum anti-aging protein klotho level in humans. To a certain extent, this provides some novel and easy-to-practice insight into the resistance or delay of aging, which including proper weight control, especially visceral adiposity. Secondly, our study used a multi-ethnic, as well as a large multi-regional population based on a large population analysis from the NHANES and included a relatively large sample size of 6 252 Americans.

In the meanwhile, there are some limitations of this study. The primary limitation is that the causality between VAI and klotho protein cannot be determined, because of the characteristics of the cross-sectional study design. In other words, visceral adiposity may make the protein klotho level go down, but lower level of this protein may lead to obesity. However, there is indeed a convincing relationship between them. Within a certain range, a lower level of protein was associated with high VAI scores. Additionally, information about VAI came from questionnaires. Some participants might be reluctant to answer the relevant questions for a variety of reasons, resulting in no answers, while others might have omitted some information when answering the questionnaire, both of which could inevitably lead to bias. Finally, there were still potentially confounding factors which were not adjusted, leading to this affecting the association between them.

## Conclusion

Based on the nationally representative population, this study demonstrated a non-linear association and a dose–response relationship between VAI and serum anti-aging protein klotho in American adults. Indeed, when VAI was less than 3.21, serum levels of klotho protein shown a significant downward trend as VAI increased, and these individuals were more likely to develop aging-related syndromes. These results indicated that careful control of adiposity may have anti-aging and health benefits by increasing serum klotho concentrations. Future large and well-designed prospective studies are warranted to confirm the causal relationship and detailed mechanisms.

## Data availability statement

The original contributions presented in the study are included in the article/supplementary material. Further inquiries can be directed to the corresponding authors.

## Ethics statement

All the study protocol included here obtained written informed consent, and were approved by the Research Ethics Review Board at the National Center for Health Statistics.

## Author contributions

Conceptualization and methodology: JiaW, YB, JC. Data acquisition: JC, ZY, JiahW. Data analysis and interpretation: JC, JiahW, ZY, SY, YX. Writing – original draft: JC. Writing – review & editing: all authors. Data curation and supervision: JC, YB. All authors contributed to the article and approved the submitted version.
